# Supplementary material for: Integration of pathologic characteristics, genetic risk and lifestyle exposure for colorectal cancer survival assessment
Source: Nat Commun. 2024 Apr 8;15:3042. doi: 10.1038/s41467-024-47204-9 (PMC11002003; doi:10.1038/s41467-024-47204-9)
Supplement: Supplementary file 1 — Supplementary Information [file 41467_2024_47204_MOESM1_ESM.pdf]

Supplementary Methods

Study subjects and individual-level quality control (QC)

NJCRC and ZJCRC cohorts

For the NJCRC and ZJCRC cohorts, the cases were diagnosed and histopathologically confirmed at the hospitals, and we used a uniform individual-level QC protocol to filter the samples as follows: (i) call rate < 95%; (ii) gender discrepancies; (iii) unexpected duplicates or probable relatives based on pairwise identity by descent (PI_HAT > 0.25); and (iv) population stratification outliers according to EIGENSTRAT software.

UK Biobank cohort

In the UK Biobank cohort, the information of colorectal cancer cases was derived from record electronic linkage with the National Health Service central registers and death registries in England, Wales, and Scotland [defined by International Classification of Diseases, 10th revision (ICD-10) codes with C18-C20]. The follow-up time of colorectal cancer survival was calculated from cancer diagnosis to death or the last follow-up (February 14, 2018). After individual-level quality control: (i) removed individuals with prevalent cancer (except non-melanoma skin cancer, based on the ICD-10 code with C44) at baseline; (ii) sex discordance; (iii) outliers for genotype missingness or excess heterozygosity; (iv) retained unrelated participants; (v) restricted to "white British" participants individuals of European (EUR) ancestry and (vi) removed individuals who decided not to participate in this program, a total of 2,621 incident colorectal cancer cases were retained for analysis.

TCGA cohort

For the TCGA cohort, the clinical data were derived from the data portal of the Genomic Data Commons (GDC), we included colorectal cancer patients based on the

following filtering criteria: (i) gender discrepancies; (ii) unexpected duplicates or probable relatives based on pairwise identity by descent (PI_HAT > 0.25); and (iii) restricted to individuals of EUR ancestry [1].

PLCO cohort

In the PLCO cohort, the incident colorectal cancers and deaths were ascertained primarily by an active, trial-led process that involved mailing annual study update questionnaires to participants, obtaining and abstracting medical records pertaining to cancer using certified tumour registrars, and obtaining death certificates to confirm mortality. Based on the following criteria: (i) excluded individuals whose outcome derived from the database of Genotypes and Phenotypes (dbGaP) was not consistent with that in the PLCO study; (ii) unexpected duplicates or probable relatives (PI_HAT > 0.25); (iii) restricted to white individuals of EUR ancestry, a total of 713 incident colorectal cancer patients remained in the following analysis.

Genotyping and imputation

NJCRC and ZJCRC cohorts

The Genomic DNA was derived from EDTA-venous blood by using the Qiagen Blood Kit (Qiagen). Genotyping was conducted using Illumina Human Omni ZhongHua Bead Chips for NJCRC cohort, and Illumina Asian Screening Array (ASA) for ZJCRC cohort. Furthermore, we imputed the non-genotyped SNPs based on the 1000 Genomes Project (Phase 1) using IMPUTE2. GTOOL was used to convert imputed data into PLINK format with a threshold of 0.9.

UK Biobank cohort

All samples were genotyped using the UK BiLEVE Axiom Array (807,411 markers tested for 49,950 participants) or UK Biobank Axiom Array (825,927 markers

tested for 438,427 participants) by Affymetrix. The genotyping data were imputed using SHAPEIT3 and IMPUTE3 based on the reference panels of HRC, UK10K and 1000 Genomes Project (Phase 3). The study protocol and information about data access are available online (http://www.ukbiobank.ac.uk/wp-content/uploads/2011/11/UK-Biobank-Protocol.pdf) and more details of the recruitment and study design have been published in previous studies [2].

TCGA cohort

We obtained access to the raw genotype data from normal blood or normal tissue samples in the TCGA database (https://tcga-data.nci.nih.gov/tcga/), through the dbGaP accession phs000178.v11.p8, which included 906,600 SNPs using the Affymetrix SNP 6.0 array. We subsequently imputed the non-genotyped SNPs based on the 1000 Genomes Project (Phase 1) using IMPUTE2. The detailed information was reported in our previous study [3].

PLCO cohort

Sequential blood samples were collected from participants assigned to the screening arm. Ninety-three percent of participants assigned to the screening arm provided a baseline blood sample. In the observational (control) arm, buccal cells were collected via mail using the “swish-and-spit” protocol, and the participation rate was 65%. A detailed description of the PLCO study is available online (http://dcp.cancer.gov/plco). We accessed the PLCO genotype data from the dbGaP (phs001286.v1.p1; phs001415.v1.p1; phs001078.v1.p1 and phs001554.v1.p1) [4,5]. Qualified genotypes for each chromosome were phased with SHAPEIT2. Non-genotyped SNPs were imputed based on haplotypes derived from the 1000 Genomes Project and the HRC reference panel. The detailed information can be found in our previous study [6].

Functional characteristics of genome-wide significant SNPs

We performed functional annotation for the prognostic loci using HaploReg v4.1 (http://archive.broadinstitute.org/mammals/haploreg/haploreg.php), RegulomeDB (http://regulome.stanford.edu/) and SNPinfo Web Server (http://snpinfo.niehs.nih.gov/). Besides, to examine predicted functional impact, we annotated variants with the CADD score (Phred scores >20 predicted as deleterious, https://cadd.gs.washington.edu/score). Furthermore, we performed expression quantitative trait loci (eQTL) analysis to evaluate the effects of significant variants on the expression of their nearby genes (within ±1 Mb region) using the data of normal Colon-Sigmoid tissues and Colon-Transverse tissues from the Genotype-Tissue Expression (GTEx, https://www.gtexportal.org/home/).

Calculation of polygenic prognostic score (PPS)

Clumping and P value thresholding

The clumping and *P* value thresholding (*i.e.*, C+T) approach, as a classic method, is used to calculate PPS using a subset of partially independent (*i.e.*, clumped) SNPs exceeding a specific GWAS association *P* value threshold [7,8]. With the combined NJCRC and UK Biobank dataset as linkage disequilibrium (LD) reference panel, leveraging the summary statistics of EAS-EUR meta-analysis for candidate SNPs (LD $r^2 < 0.1$), we used PLINK software (version 1.90) to obtain three subsets of variants, where we set the region size to be 500 kb, with different *P* value thresholds (*i.e.*, 1.00×10^{-5}, 1.00×10^{-4}, and 0.001).

LASSO

The least absolute shrinkage and selection operator (LASSO) is a popular

penalized regression method used in high dimensional data to prevent overfitting, with an L1 penalty to shrink some regression coefficients to zero [9,10]. The larger the value of penalty parameter lambda (λ, *i.e.*, tuning parameter), fewer predictors will be selected. We adopted a penalized Cox regression model with LASSO penalty to achieve shrinkage and variable selection simultaneously, with ten-fold cross validations for determining the optimal values of lambda, implemented by R package *glmnet*. The optimal lambda was selected via 1-standard error (SE) criteria, to determine included SNPs for PPS construction. Finally, we constructed two LASSO-based PPSs based on the weights derived from meta-analysis or the LASSO penalized regression.

Random survival forest (RSF)

The RSF method, an extension of Breiman's random forest, which obtains bootstrap samples from the original cohort, and then grows a tree for each bootstrapped sample on the basis of a splitting rule applied to a tree node to maximize survival differences across daughter nodes [11]. The process is repeated numerous times (number of trees = 2,000 in this study) so that a forest of trees is created. The importance of each variable was determined by variable importance (VIMP), derived from the difference between the out-of-bag (OOB) c-indexes of the original OOB data and that of the permuted OOB data, where variables with larger VIMP are considered more predictive, implemented by R package *randomForestSRC*. We constructed candidate PPSs by adding SNPs in the decremental order of VIMP, and the PPS with the highest 5-year AUC in the validation dataset (*i.e.*, TCGA cohort) was determined as optimal.

CoxBoost

The CoxBoost method, a likelihood-based boosting algorithm in the Cox proportional hazards model [12]. Likelihood-based boosting usually uses base learners that maximise an overall likelihood in each boosting step, which selects only the base-

learner with the largest increasement in the likelihood. CoxBoost is used for models with numerical predictors and allows for mandatory covariates with unpenalized parameter estimates. We adopted a boosted Cox regression model in feature selection, with ten-fold cross validations for determining the optimal boosting steps, implemented by R package *CoxBoost*. After identifying included SNPs for PPS construction, we constructed two CoxBoost-based PPSs based on the weights derived from meta-analysis or the boosted regression.

Supplementary Table 1. Summary of five lifestyle factors in the PLCO cohort.

Lifestyle factor[a]	Definition[b]	All cases (N = 713)	Death (N = 177)	Survival (N = 536)
BMI				
1	18.5-24.9 kg/m^3	202	55	147
0	-	492	119	373
Missing		19	3	16
Smoking status				
1	Never	311	73	238
0	-	402	104	298
Drinking status				
1	Never	52	13	39
0	-	577	140	437
Missing		84	24	60
Red and processed meat intake				
1	< 90g/day	415	96	319
0	-	237	62	175
Missing		61	19	42
Vegetable and fruit intake				
1	> 400g/day	414	98	316
0	-	238	60	178
Missing		61	19	42

[a] Each lifestyle factor was given a score of 0 or 1, with 1 representing the healthy category.
[b] Definition of healthy behaviour category.
Note: BMI, body mass index; PLCO, Prostate, Lung, Colorectal and Ovarian Cancer Screening Trial.

Supplementary Table 2. Summary of two suggestive genome-wide significant loci associated with colorectal cancer overall survival.

Chr	SNP	Position[a]	Allele[b]	Cohort[c]	RAF[d]	HR (95% CI)[e]	P^{e}	I^2	P_{het}^{f}
9	rs10967103	25872263	T/C	NJCRC	0.046	1.60 (1.16, 2.21)	0.004		
				UK Biobank	0.017	1.81 (1.31, 2.49)	2.93E-04		
				Combined		1.70 (1.36, 2.13)	4.05E-06	0	0.606
12	rs79067806	40209445	G/A	NJCRC	0.014	2.20 (1.36, 3.57)	0.001		
				UK Biobank	0.016	1.77 (1.27, 2.46)	6.74E-04		
				Combined		1.89 (1.44, 2.49)	4.14E-06	0	0.464

[a] Chromosomal position, hg19/GRCh37 build.
[b] Risk/reference allele.
[c] NJCRC: East Asian ancestry; UK Biobank: European ancestry.
[d] Risk allele frequency.
[e] Derived from Cox regression model, with the adjustment of corresponding covariates (NJCRC cohort: sex, age, smoking status, drinking status, stage, grade and top 10 principal components; UK Biobank cohort: sex, age, BMI, smoking status, drinking status and top 10 principal components). Combined results were obtained from Meta-analysis. The *P* value is two-sided.
[f] *P* value for heterogeneity test. The *P* value is two-sided.
Note: BMI, body mass index.

Supplementary Table 3. Functional annotation for the two suggestive genome-wide survival-associated significant loci.

Locus	SNP	Nearby gene	Regulome DB score[a]	CADD (Phred)[b]	Allele[c]	OR (95% CI)[d]	*P*[d]
9p21.2	rs10967103	*TUSC1*	5	0.177	T/C	1.02 (0.96, 1.09)	0.449
12q12	rs79067806	*SLC2A13*	5	9.289	G/A	1.00 (0.94, 1.07)	0.955

[a] Regulome DB Score: 5, TF binding or DNase peak.
[b] CADD, Combined Annotation Dependent Depletion.
[c] Risk/reference allele.
[d] The association of two loci with colorectal cancer risk, derived from Meta-analysis of colorectal cancer GWAS in East Asian and European population. The *P* value is two-sided.

Supplementary Table 4. Validation of the optimal polygenic prognostic score (*i.e.*, PPS_{287}) in the ZJCRC and PLCO cohorts.

Cohort	PPS	All follow-up		3-year follow-up			5-year follow-up		
		HR (95% CI)[a]	*P*[a]	HR (95% CI)[a]	*P*[a]	AUC[b]	HR (95% CI)[a]	*P*[a]	AUC[b]
ZJCRC	Per SD	1.90 (1.61, 2.24)	3.21E-14	1.97 (1.55, 2.50)	2.77E-08	0.694	1.80 (1.49, 2.18)	8.21E-10	0.649
	Low (N = 272)	1.00 (reference)		1.00 (reference)			1.00 (reference)		
	High (N = 271)	3.24 (2.27, 4.63)	1.05E-10	3.90 (2.25, 6.77)	1.23E-06		2.63 (1.77, 3.90)	1.49E-06	
PLCO	Per SD	1.80 (1.49, 2.17)	1.11E-09	1.62 (1.27, 2.08)	1.38E-04	0.641	1.68 (1.36, 2.09)	1.88E-06	0.658
	Low (N = 357)	1.00 (reference)		1.00 (reference)			1.00 (reference)		
	High (N = 356)	2.25 (1.54, 3.28)	2.72E-05	1.83 (1.12, 2.99)	0.016		2.13 (1.38, 3.27)	5.74E-04	

[a] Derived from cox regression model, with the adjustment of corresponding factors (ZJCRC: sex, age, smoking status, drinking status and top 10 principal components; PLCO: sex, age, smoking status, drinking status, research center, arm, stage, grade and top 10 principal components). The *P* value is two-sided.

[b] Area under the time-dependent ROC curve.

Note: PPS, polygenic prognostic score; PLCO, Prostate, Lung, Colorectal and Ovarian Cancer Screening Trial; HR, hazard ratio; 95% CI, 95% confidence interval; ROC, receiver operating characteristics; SD, standard deviation.

Supplementary Table 5. Sensitivity analysis for the association between polygenic prognostic score (*i.e.*, PPS_{287}) and colorectal cancer overall survival in the ZJCRC and PLCO cohorts.

Sensitivity analysis	Cohort	PPS	All follow-up		3-year follow-up		5-year follow-up	
			HR (95% CI)[a]	*P*[a]	HR (95% CI)[a]	*P*[a]	HR (95% CI)[a]	*P*[a]
Excluded colorectal cancer patients who died within the first year of follow-up	ZJCRC	Per SD	1.91 (1.61, 2.27)	2.61E-13	2.04 (1.56, 2.66)	1.65E-07	1.82 (1.49, 2.22)	5.53E-09
		Low (N = 268)	1.00 (reference)		1.00 (reference)		1.00 (reference)	
		High (N = 261)	3.27 (2.25, 4.75)	5.31E-10	4.28 (2.29, 7.99)	5.14E-06	2.60 (1.71, 3.95)	7.37E-06
	PLCO	Per SD	1.86 (1.5, 2.32)	2.30E-08	1.43 (1.03, 2.00)	0.035	1.63 (1.26, 2.12)	2.18E-04
		Low (N = 337)	1.00 (reference)		1.00 (reference)		1.00 (reference)	
		High (N = 322)	2.38 (1.53, 3.71)	1.20E-04	1.51 (0.77, 2.98)	0.229	2.07 (1.22, 3.52)	0.007
Ancestry-corrected PPS	ZJCRC	Per SD	1.83 (1.55, 2.14)	1.90E-13	1.93 (1.53, 2.44)	2.44E-08	1.77 (1.47, 2.13)	1.22E-09
		Low (N = 272)	1.00 (reference)		1.00 (reference)		1.00 (reference)	
		High (N = 271)	2.78 (1.97, 3.92)	6.41E-09	3.97 (2.30, 6.85)	7.13E-07	2.49 (1.69, 3.67)	3.95E-06
	PLCO	Per SD	1.75 (1.46, 2.09)	1.60E-09	1.57 (1.24, 1.98)	1.70E-04	1.65 (1.34, 2.02)	1.68E-06
		Low (N = 357)	1.00 (reference)		1.00 (reference)		1.00 (reference)	
		High (N = 356)	2.29 (1.57, 3.34)	1.60E-05	1.89 (1.16, 3.08)	0.011	2.22 (1.45, 3.41)	2.57E-04

[a] Derived from cox regression model, with the adjustment of corresponding factors (ZJCRC: sex, age, smoking status, drinking status and top 10 principal components; PLCO: sex, age, smoking status, drinking status, research center, arm, stage, grade and top 10 principal components) when appropriate. The *P* value is two-sided.

Note: PPS, polygenic prognostic score; PLCO, Prostate, Lung, Colorectal and Ovarian Cancer Screening Trial; HR, hazard ratio; 95% CI, 95% confidence interval; SD, standard deviation.

Supplementary Table 6. Univariate and multivariate analysis for the association of traditional risk factors and polygenic prognostic score with colorectal cancer overall survival in the ZJCRC cohort.

Variable	Death (n = 152)	Survival (n = 391)	Univariate[a]		Multivariate[a]	
			HR (95% CI)	*P*	HR (95% CI)	*P*
Sex, N						
Male	86	203	1.00 (reference)		1.00 (reference)	
Female	66	188	0.86 (0.62, 1.19)	0.371	0.90 (0.57, 1.43)	0.659
Age (year), mean ± SD	67.65 ± 11.05	61.93 ± 10.22	1.05 (1.04, 1.07)	8.33E-10	1.06 (1.04, 1.08)	9.80E-11
Smoking status, N						
Ever	58	127	1.21 (0.87, 1.69)	0.265	1.56 (0.97, 2.52)	0.065
Never	93	263	1.00 (reference)		1.00 (reference)	
Missing	1	1				
Drinking status, N						
Ever	36	101	0.82 (0.56, 1.20)	0.316	0.59 (0.38, 0.93)	0.022
Never	115	288	1.00 (reference)		1.00 (reference)	
Missing	1	2				
PPS, N						
Low	48	224	1.00 (reference)		1.00 (reference)	
High	104	167	2.81 (1.98, 3.98)	6.23E-09	3.24 (2.27, 4.63)	1.05E-10

[a] Derived from the Cox regression model, with the additional adjustment of top 10 principal components. The *P* value is two-sided.
Note: PPS, polygenic prognostic score.

Supplementary Table 7. Univariate and multivariate analysis for the association of traditional risk factors and polygenic prognostic score with colorectal cancer overall survival in the PLCO cohort.

Variable	Death (n = 177)	Survival (n = 536)	Univariate[a]		Multivariate[a]	
			HR (95% CI)	*P*	HR (95% CI)	*P*
Sex, N						
Male	103	316	1.00 (reference)		1.00 (reference)	
Female	74	220	0.97 (0.71, 1.32)	0.836	1.00 (0.69, 1.47)	0.988
Age (year), mean ± SD	70.19 ± 6.45	70.05 ± 6.67	1.05 (1.03, 1.08)	5.21E-05	1.04 (1.01, 1.07)	0.016
Smoking status, N						
Ever	104	298	1.12 (0.82, 1.53)	0.463	1.69 (1.14, 2.51)	0.010
Never	73	238	1.00 (reference)		1.00 (reference)	
Drinking status, N						
Ever	140	437	1.21 (0.64, 2.30)	0.552	1.01 (0.51, 2.01)	0.980
Never	13	39	1.00 (reference)		1.00 (reference)	
Missing	24	60				
Stage[b], N			2.82 (2.39, 3.33)[d]	4.69E-34[d]	2.71 (2.20, 3.33)[d]	8.21E-21[d]
1	38	232	1.00 (reference)		1.00 (reference)	
2	30	165	1.54 (0.95, 2.50)	0.082	1.36 (0.76, 2.43)	0.294
3	41	113	2.77 (1.76, 4.37)	1.04E-05	2.40 (1.40, 4.11)	0.001
4	67	20	26.15 (16.4, 41.69)	8.30E-43	24.28 (13.59, 43.4)	4.96E-27
Missing	1	6				
Grade[c], N			2.53 (1.93, 3.33)[d]	2.48E-11[d]	1.53 (1.12, 2.09)[d]	0.007[d]
G1	8	55	1.00 (reference)		1.00 (reference)	
G2	99	372	1.91 (0.92, 3.95)	0.083	1.84 (0.73, 4.65)	0.198
G3	45	74	5.20 (2.42, 11.18)	2.39E-05	2.97 (1.10, 8.02)	0.032
G4	6	3	12.86 (4.10, 40.40)	1.21E-05	4.52 (1.11, 18.44)	0.035

Missing	19	32				
PPS, N						
Low	63	294	1.00 (reference)		1.00 (reference)	
High	114	242	1.92 (1.41, 2.63)	4.15E-05	2.25 (1.54, 3.28)	2.72E-05

[a] Derived from the Cox regression model, with the additional adjustment of arm, research center and top 10 principal components. The *P* value is two-sided.
[b] Combined clinical and pathologic stage (stage I, stage II, stage III and stage IV) for PLCO cohort.
[c] G1, well differentiated; G2, moderately differentiated; G3, poorly differentiated; G4, undifferentiated.
[d] Trend analysis.
Note: PPS, polygenic prognostic score; PLCO, Prostate, Lung, Colorectal and Ovarian Cancer Screening Trial.

Supplementary Table 8. Performance comparison regarding 5-year survival prediction of different colorectal cancer prognostic models in the ZJCRC and PLCO cohorts.

Model[a]	ZJCRC cohort			PLCO cohort		
	AUC[b]	95% CI[c]	*P*[c]	AUC[b]	95% CI[c]	*P*[c]
PPS	0.649	0.592, 0.705	< 0.01	0.658	0.612, 0.714	< 0.01
Traditional	0.644	0.588, 0.710	< 0.01	0.807	0.751, 0.868	< 0.01
Combined	0.699	0.649, 0.762	Reference	0.834	0.794, 0.886	Reference

[a] The traditional model included sex, age, smoking status and drinking status in the ZJCRC cohort; sex, age, smoking status, drinking status, stage and grade in the PLCO cohort. The combined model included traditional factors and PPS.

[b] AUC at 5-year survival.

[c] Derived from bootstrap method with 10,000 iterations. The *P* value is two-sided.

Note: PLCO, Prostate, Lung, Colorectal and Ovarian Cancer Screening Trial; PPS, polygenic prognostic score; ROC, receiver operating characteristics; AUC, area under the curve.

Supplementary Table 9. Summary of the performance of traditional and combined prognostic models in the ZJCRC and PLCO cohorts.

Cohort	Metric	Traditional model[a]	Combined model[a]
ZJCRC	AUC (95% CI)[b]	0.644 (0.588, 0.710)	0.699 (0.649, 0.762)
	Sensitivity (95% CI)[b]	0.642 (0.552, 0.695)	0.635 (0.567, 0.737)
	Specificity (95% CI)[b]	0.601 (0.552, 0.691)	0.673 (0.602, 0.746)
	Harrell's C index (95% CI)	0.652 (0.603, 0.700)	0.715 (0.670, 0.760)
	R^2_D (%, 95% CI) [c]	17.92% (10.06%, 26.48%)	31.89% (23.18%, 40.15%)
PLCO	AUC (95% CI)[b]	0.807 (0.751, 0.868)	0.834 (0.794, 0.886)
	Sensitivity (95% CI)[b]	0.696 (0.623, 0.806)	0.745 (0.654, 0.860)
	Specificity (95% CI)[b]	0.780 (0.667, 0.850)	0.760 (0.657, 0.861)
	Harrell's C index (95% CI)	0.786 (0.745, 0.827)	0.818 (0.785, 0.852)
	R^2_D (%, 95% CI) [c]	47.23% (37.80%, 55.28%)	51.22% (42.94%, 58.21%)

[a] The traditional model included sex, age, smoking status and drinking status in the ZJCRC cohort; sex, age, smoking status, drinking status, stage and grade in the PLCO cohort. The combined model included traditional factors and PPS.

[b] AUC, sensitivity and specificity at 5-year survival, of which the optimal sensitivity and specificity were selected based on the Index of Union (IU) method. The 95% CIs were derived from bootstrap method with 10,000 iterations.

[c] Royston and Sauerbrei's R^2_D in Cox proportional hazards models.

Note: PLCO, Prostate, Lung, Colorectal and Ovarian Cancer Screening Trial; PPS, polygenic prognostic score; ROC, receiver operating characteristics; AUC, area under the curve; 95% CI, 95% confidence interval.

Supplementary Table 10. Univariate and multivariate analysis for the association of pathologic stage or grade, genetic risk and healthy lifestyle with colorectal cancer overall survival in the PLCO cohort.

Variable	Death (n = 177)	Survival (n = 536)	Univariate[a]		Multivariate[a]	
			HR (95% CI)	*P*	HR (95% CI)	*P*
Stage/grade[b], N						
Low	56	339	1.00 (reference)		1.00 (reference)	
High	115	168	4.48 (3.21, 6.26)	1.42E-18	4.78 (3.28, 6.97)	3.77E-16
Missing	6	29				
Genetic risk[c], N						
Low	63	294	1.00 (reference)		1.00 (reference)	
High	114	242	1.88 (1.38, 2.58)	7.47E-05	1.97 (1.38, 2.81)	1.71E-04
Lifestyle[d], N						
Unfavourable	53	143	1.00 (reference)		1.00 (reference)	
Favourable	97	319	0.77 (0.53, 1.11)	0.167	0.73 (0.50, 1.07)	0.106
Missing	27	74				

[a] Derived from the Cox regression model, with the additional adjustment of sex, age, arm, research center and top 10 principal components. The *P* value is two-sided.

[b] Low: low stage (stage I and stage II) and low grade (G1 and G2); High: high stage (stage III and stage IV) or high grade (G3 and G4).

[c] Low: low PPS; High: high PPS.

[d] Unfavourable: 0 and 1 healthy lifestyle score; Favourable: with ⩾ 2 healthy lifestyle score.

Note: PPS, polygenic prognostic score; PLCO, Prostate, Lung, Colorectal and Ovarian Cancer Screening Trial.

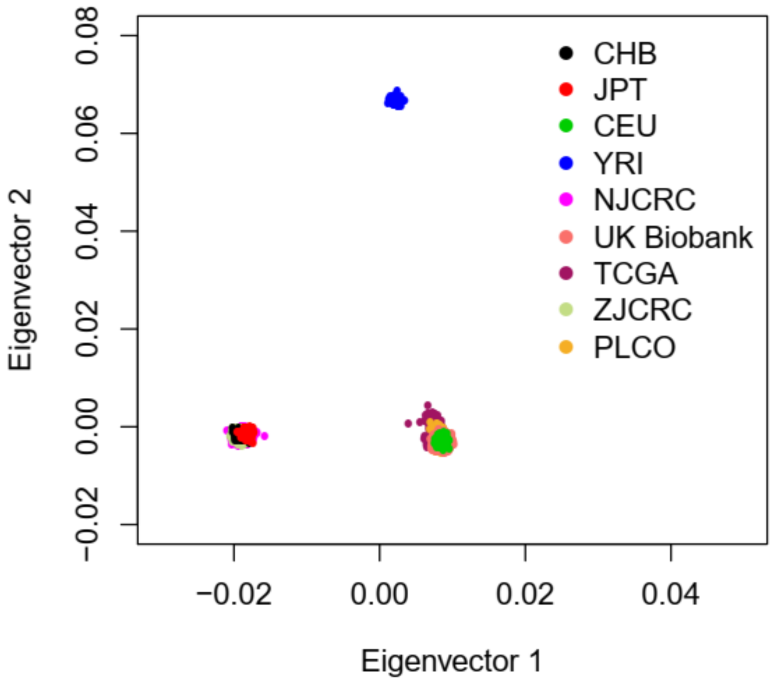

Supplementary Figure 1. Principal component analysis (PCA) for the five cohorts (discovery stage: NJCRC and UK Biobank cohorts; validation stage: TCGA cohort; testing stage: ZJCRC and PLCO cohorts) of colorectal cancer patients and 1000 Genomes Project populations.

Note: CHB, Han Chinese in Beijing, China; JPT, Japanese in Tokyo, Japan; CEU, Utah residents with Northern and Western European ancestry; YRI, Yoruba in Ibadan, Nigeria; TCGA, The Cancer Genome Atlas; PLCO, Prostate, Lung, Colorectal and Ovarian Cancer Screening Trial.

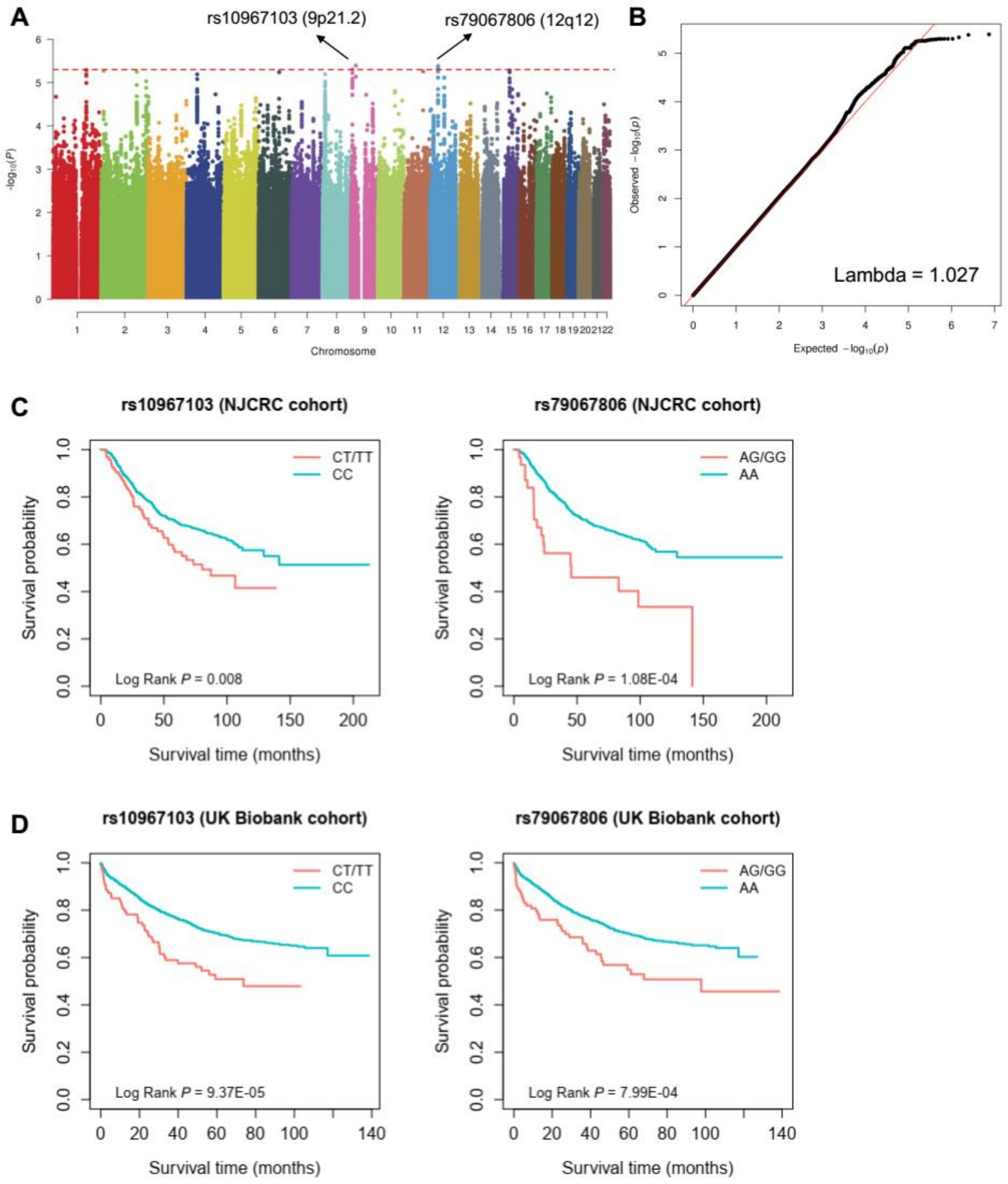

Supplementary Figure 2. Summary of the colorectal cancer overall survival GWAS meta-analysis from the NJCRC and UK Biobank cohorts. (A) Manhattan plot for the meta-analysis. The associations (-log10 (*P*-value) values, Y-axis) are plotted against genomic position (X-axis by chromosome and the chromosomal position of NCBI build 37). The red dashed line indicates the suggestive genome-wide significance threshold (*P* = 5E-06). (B) Quantile-quantile plot and genomic inflation factor for the meta-analysis. The X axis shows the expected distribution of the observed -log10 (*P* values)

under the null hypothesis of no association. The Y axis shows the distribution of the observed -log10 (*P* values) of meta-analysis. (C-D) Kaplan-Meier curves for overall survival probability stratified by rs10967103 and rs79067806 in the NJCRC and UK Biobank cohorts.

Note: GWAS, genome-wide association study.

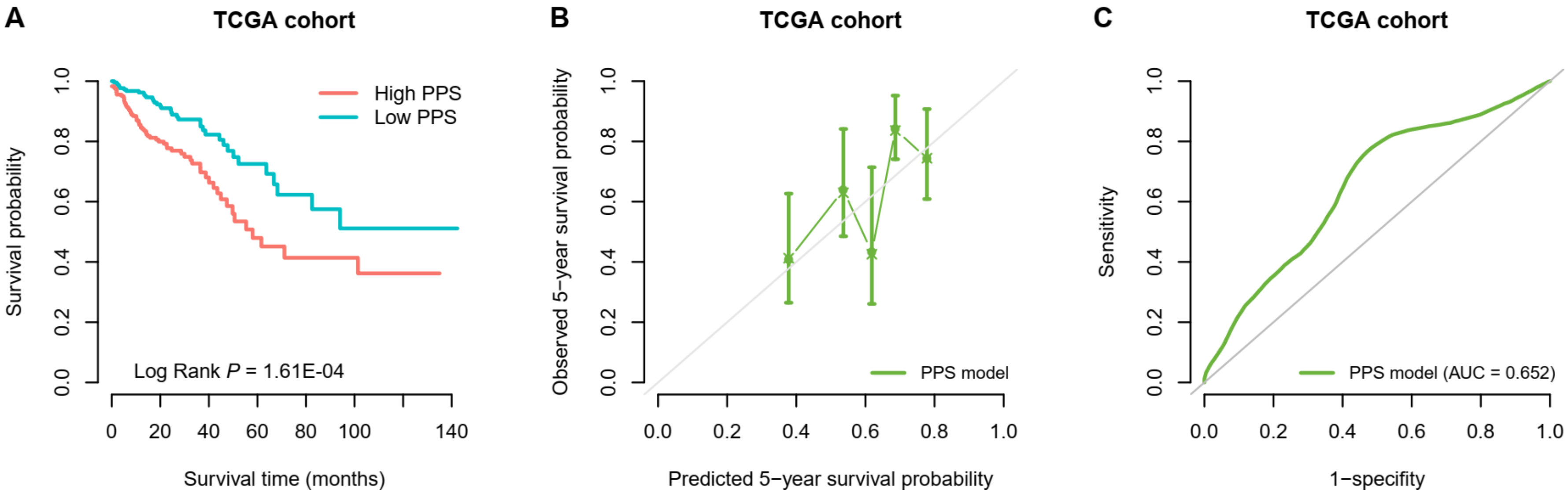

Supplementary Figure 3. Prognostic evaluation of the optimal polygenic prognostic score (*i.e.*, PPS_{287}) in the TCGA cohort. (A) Kaplan-Meier curves for overall survival probability stratified by different levels of PPS (based on median value). (B) Calibration curve of PPS model for predicting 5-year survival probability. The vertical error bars denote the 95% CI. (C) Time-dependent ROC curves of PPS models regarding 5-year survival probability. The sample size of TCGA cohort is 470 cases.

Note: TCGA, The Cancer Genome Atlas; PPS, polygenic prognostic score; ROC, receiver operating characteristics; AUC, area under the curve; ; 95% CI, 95% confidence interval.

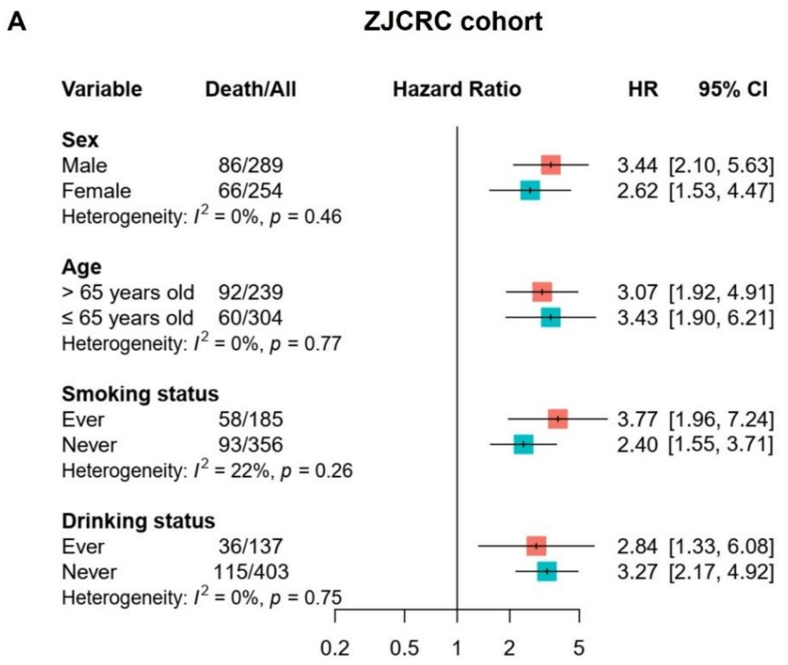

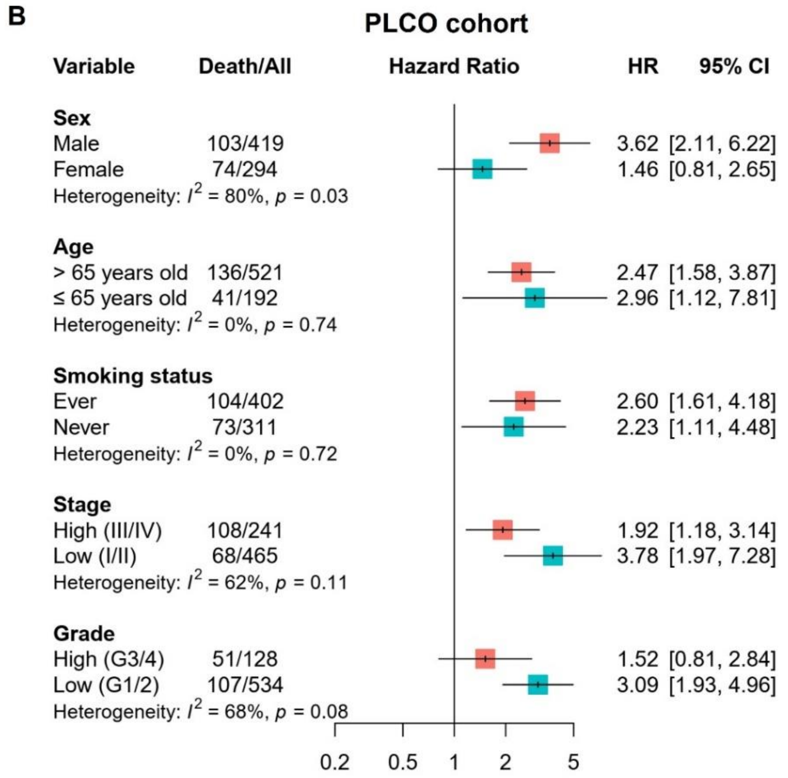

Supplementary Figure 4. The association between polygenic prognostic score (*i.e.*, PPS_{287}, high *vs.* low) and colorectal cancer overall survival stratified by different clinical factors in the (A) ZJCRC and (B) PLCO cohorts. The horizontal error bars denote the 95% CI. The sample sizes of ZJCRC and PLCO cohorts are 543 and 713 cases. Note: PLCO, Prostate, Lung, Colorectal and Ovarian Cancer Screening Trial.

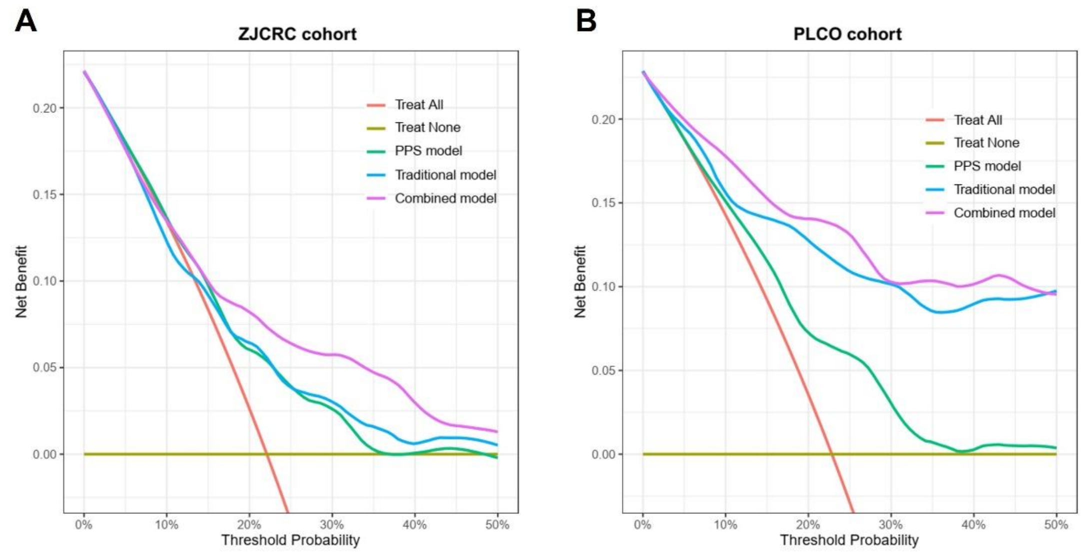

Supplementary Figure 5. Decision curve analysis for different colorectal cancer prognostic models in the (A) ZJCRC and (B) PLCO cohorts. The figures show net benefit at 5 years of follow-up. The sample sizes of ZJCRC and PLCO cohorts are 543 and 713 cases.

Note: PLCO, Prostate, Lung, Colorectal and Ovarian Cancer Screening Trial; PPS, polygenic prognostic score.

References:

1. Carrot-Zhang, J., *et al.* Comprehensive Analysis of Genetic Ancestry and Its Molecular Correlates in Cancer. *CANCER CELL* **37**, 639-654 (2020).
2. Bycroft, C., *et al.* The UK Biobank resource with deep phenotyping and genomic data. *NATURE* **562**, 203-209 (2018).
3. Xin, J., *et al.* Systematic evaluation of the effects of genetic variants on PIWI-interacting RNA expression across 33 cancer types. *NUCLEIC ACIDS RES* **49**, 90-97 (2021).
4. Black, A., *et al.* PLCO: Evolution of an Epidemiologic Resource and Opportunities for Future Studies. *REV RECENT CLIN TRIA* **10**, 238-245 (2015).
5. Zhu, C.S., *et al.* The prostate, lung, colorectal, and ovarian cancer screening trial and its associated research resource. *JNCI-J NATL CANCER I* **105**, 1684-1693 (2013).
6. Chu, H., *et al.* A prospective study of the associations among fine particulate matter, genetic variants, and the risk of colorectal cancer. *ENVIRON INT* **147**, 106309 (2021).
7. Xin, J., *et al.* Risk assessment for colorectal cancer via polygenic risk score and lifestyle exposure: a large-scale association study of East Asian and European populations. *GENOME MED* **15**, 4 (2023).
8. Choi, S.W., Mak, T.S. & O'Reilly, P.F. Tutorial: a guide to performing polygenic risk score analyses. *NAT PROTOC* **15**, 2759-2772 (2020).
9. Tibshirani, R. The lasso method for variable selection in the Cox model. *STAT MED* **16**, 385-395 (1997).
10. Wei, J.H., *et al.* Predictive value of single-nucleotide polymorphism signature for recurrence in localised renal cell carcinoma: a retrospective analysis and multicentre validation study. *LANCET ONCOL* **20**, 591-600 (2019).
11. Hemant, I., Udaya, B.K., Eugene, H.B. & Michael, S.L. Random survival forests. *The Annals of Applied Statistics* **2**, 841-860 (2008).
12. Tutz, G. & Binder, H. Generalized additive modeling with implicit variable selection by likelihood-based boosting. *BIOMETRICS* **62**, 961-971 (2006).
