# Supplementary material for: Integration of pathologic characteristics, genetic risk and lifestyle exposure for colorectal cancer survival assessment
Source: Nat Commun. 2024 Apr 8;15:3042. doi: 10.1038/s41467-024-47204-9 (PMC11002003; doi:10.1038/s41467-024-47204-9)
Supplement: Supplementary file 4 — Description of Additional Supplementary Files [file 41467_2024_47204_MOESM4_ESM.pdf]

Description of Additional Supplementary Files

File name: Supplementary Data 1

Description: Summary of the 287 variants for polygenic prognostic score construction.
